# Cost-effectiveness of measles control during elimination in Ontario, Canada, 2015

**DOI:** 10.2807/1560-7917.ES.2019.24.11.1800370

**Published:** 2019-03-14

**Authors:** Lauren C Ramsay, Natasha S Crowcroft, Shari Thomas, Elena Aruffo, Alexandra Teslya, Jane M Heffernan, Effie Gournis, Joanne Hiebert, Valerie Jaeger, Manisa Jiaravuthisan, Jennifer Sharron, Alberto Severini, Shelley L Deeks, Jonathan Gubbay, Tony Mazzulli, Beate Sander

**Affiliations:** 1 Public Health Ontario, Toronto, Ontario, Canada; 2 University of Toronto, Toronto, Ontario, Canada; 3 University Health Network, Eaton Building, Toronto, Ontario, Canada; 4 York University, Toronto, Ontario, Canada; 5 Toronto Public Health, Toronto, Ontario, Canada; 6 Public Health Agency of Canada, Winnipeg, Manitoba, Canada; 7 Niagara Region Public Health, Thorold, Ontario, Canada; 8 University of Manitoba, Winnipeg, Manitoba, Canada; 9 Institute for Clinical Evaluative Sciences, Toronto, Ontario, Canada

**Keywords:** measles, public health policy, modelling, economic evaluation, Canada

## Abstract

**Background:**

Given that measles is eliminated in Canada and measles immunisation coverage in Ontario is high, it has been questioned whether Ontario’s measles outbreak response is worthwhile.

**Aim:**

Our objective was to determine cost-effectiveness of measles containment protocols in Ontario from the healthcare payer perspective.

**Methods:**

We developed a decision-analysis model comparing Ontario’s measles containment strategy (based on actual 2015 outbreak data) with a hypothetical ‘modified response’. The modified scenario assumed 10% response costs with reduced case and contact tracing and no outbreak-associated vaccinations; it was based on local and provincial administrative and laboratory data and parameters from peer-reviewed literature. Short- and long-term health outcomes, quality-adjusted life years (QALYs) and costs discounted at 1.5%, were estimated. We conducted one- and two-way sensitivity analyses.

**Results:**

The 2015 outbreak in Ontario comprised 16 measles cases and an estimated 3,369 contacts. Predictive modelling suggested that the outbreak response prevented 16 outbreak-associated cases at a cost of CAD 1,213,491 (EUR 861,579). The incremental cost-effectiveness ratio was CAD 739,063 (EUR 524,735) per QALY gained for the outbreak response vs modified response. To meet the commonly accepted cost-effectiveness threshold of CAD 50,000 (EUR 35,500) per QALY gained, the outbreak response would have to prevent 94 measles cases. In sensitivity analyses, the findings were robust.

**Conclusions:**

Ontario’s measles outbreak response exceeds generally accepted cost-effectiveness thresholds and may not be the most efficient use of public health resources from a healthcare payer perspective. These findings should be balanced against benefits of increased vaccine coverage and maintaining elimination status.

## Background

Measles is a highly infectious viral infection that results in fever and maculopapular rash [1]. In severe cases it can lead to severe respiratory infection (including pneumonia) and encephalitis [1]. The last endemic case of measles in Canada was reported in 1997; in Ontario, the number of measles cases ranged from 58 cases (in 2008) to three cases (in 2012) per year over the last 10 years [2]. Endemic measles transmission is the transmission of measles cases within a geographic area that continues for more than 1 year [3]; measles transmission within a region that does not persist suggests that population immunity is high enough to limit chains of transmission. Transmission of these cases is referred to as indigenous, however, the initial measles case spurring indigenous transmission is imported from another geographic area [3]. Despite the elimination of measles in 1997, Canada continues to experience the importation of measles cases [4].

Measles outbreaks have significant economic impact [5,6], and in jurisdictions like Ontario, Canada, where measles has been eliminated [7], routine follow-up of cases and contacts requires intense response by local public health agencies (LPHA) under current protocols [8]. Given the highly infectious nature of measles, contacts can be numerous and, if required, need rapid post-exposure prophylaxis. A detailed travel history must be obtained from each case, as well as information on locations in the community where cases may have been exposed or exposed others to measles. All contacts must be notified of their exposure and evaluated to determine their susceptibility to measles. These activities can be highly resource-intensive when performed for even a small number of cases, and divert public health staff time away from other important public health activities. In addition to LPHA, several other institutions are involved in the outbreak response activities including Public Health Ontario (PHO; the provincial public health agency and provincial laboratory), Ontario Ministry of Health and Long Term Care (MOHLTC), and the National Microbiology Laboratory (NML; the federal public health agency laboratory).

During the first quarter of 2015, a measles outbreak of unknown source occurred in Ontario that has been previously described [9]. During this outbreak, a total of 18 related measles cases were identified between 25 January and 17 February 2015 [9]. The majority of outbreak cases (n = 16) were concentrated within the borders of two LPHA: Toronto Public Health (TPH) and Niagara Region Public Health (NRPH). Intense public health activity surrounding this outbreak triggered a discussion at the Council of Ontario Medical Officers of Health (COMOH) about whether routine public health control measures for measles were cost-effective given high routine coverage of close to 95% for two-dose measles vaccination [10].

This study aimed to determine the cost of measles containment in public health agencies in a Canadian jurisdiction, the benefits of measles containment, including the number of potential cases prevented, and the cost-effectiveness of measles containment compared with a modified response; it focused on the healthcare payer perspective in a setting with high measles vaccine coverage.

## Methods

In accordance with the Canadian guidelines for economic evaluation [11] this economic analysis was conducted from the perspective of the healthcare payer, estimating the impact of a modified response to a measles case in a highly immunised population. All known publicly funded healthcare costs were included regardless of whether they were borne by local municipalities (e.g. LPHA response costs), provincial government (e.g. vaccine, laboratory costs and treatment cost of measles cases) or federal government (e.g. NML). Health outcomes included the number of potentially prevented cases and quality-adjusted life years (QALYs). QALYs and costs were discounted at 0%, 1.5% and 3%. Cost-effectiveness was assessed against commonly used cost-effectiveness thresholds of CAD 50,000 (EUR 35,500) and CAD 100,000 (EUR 71,000) per QALY gained [12,13].

### Model structure

**Figure 1:**
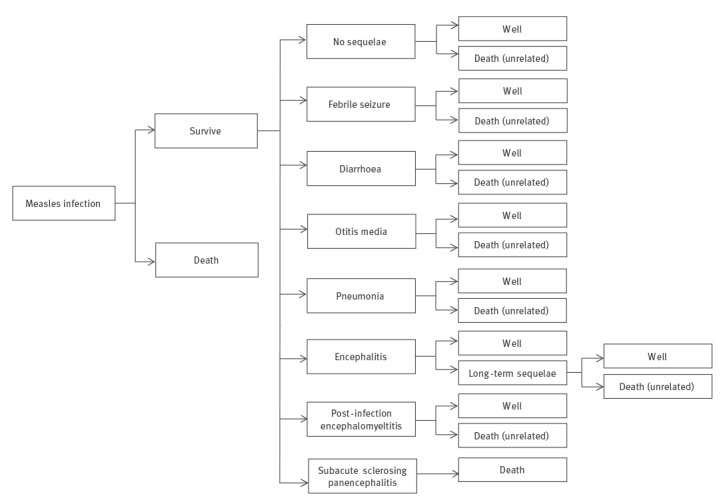
Simplified schematic of decision-analysis model, cost-effectiveness study of measles control, Ontario, Canada, 2015

A decision-analysis model was developed in Microsoft Excel to determine the cost effectiveness of measles containment strategies compared with a less intense ‘modified response’ strategy. The model incorporated acute measles infection, short-term sequelae and long-term sequelae over a lifetime time horizon (Figure 1). The model considered the population from the two Ontario outbreak regions: Toronto (n = 2,839,176) and Niagara Region (n = 447,967).

### Data

All data and sources are reported in Table 1. The analysis was conducted using real cost data from Ontario (salaries, healthcare costs etc). All costs are reported in 2015 Canadian dollars and 2015 Euros (converted using the average 2015 exchange rate: 2015 CAD 1 = 2015 EUR 0.71) [14,15]. 

**Table 1:** Input parameters, cost-effectiveness study of measles control, Ontario, Canada, 2015

Cost of intervention	Cost (CAD; EUR 2015)		Source
	Base case	Modified response	
Local Public Health Agency costs			
Toronto Public Health costs	534,270; 379,332	53,428; 37,934	TPH
Niagara Region Public Health costs	33,464; 23,759	3,347; 2,376	NRPH
Provincial and federal costs			
Public Health Ontario	48,199; 34,221	4,820; 3,422	PHO
National Microbiology Laboratory	9,118; 6,474		NML
Public Health Ontario laboratories	240,914; 171,049		PHOL
Healthcare costs (cost per visit)			
Emergency room visits	195; 138		[40]
Hospitalisations	5,298; 3,762		[41]
Outpatient visit	30; 21		[42]
Vaccine costs			
MMR doses	20; 14		Assumption based on [38]
PEP-IgG doses	100; 71		[39]
Doses	Base case	Modified response	Source
Vaccine distribution attributable to outbreak (doses)			
Toronto Public Health MMR	14,316	0	MOHLTC
Toronto Public Health PEP-IgG	13	13	TPH
Niagara Region Public Health MMR	1,613	0	MOHLTC
Niagara Region Public Health PEP-IgG	0	0	NRPH
Health outcomes	Probability		Source
Complications			
Febrile seizure (measles)	0.012		[20]
Diarrhoea (measles)	0.082		[20]
Otitis media (measles)	0.080		[21]
Pneumonia (measles)	0.035		[21]
Encephalitis (measles)	0.015		[21]
Long-term sequelae (encephalitis)	0.250		[23]
Post-infection encephalomyelitis (measles)	0.002		[20]
Subacute sclerosing panencephalitis (measles)	0.0004		[22]
Physician visit	0.775		[24]
Emergency room visit	0.250		[24]
Hospitalisation	0.192		[24]
Death	0.002		[26,27,31-34,49]
Health-related utilities			
No measles (<5 years)	0.94		[37]
No measles (5–19 years)	0.94		[37]
No measles (≥20 years)	0.92		[37]
Measles infection (<7 years)	0.92		[35]
Measles infection (7–12 years)	0.92		[35]
Measles infection (≥13 years)	0.90		[35]
Pneumonia (adolescent)	0.82		[36]
Pneumonia (adult)	0.91		[36]
Encephalitis short-term	0.51		[36]
Encephalitis long-term sequelae	0.77		[36]

IgG: immunoglobulin G; MMR: measles-mumps-rubella; NML: National Microbiology Laboratory; NRPH: Niagara Region Public Health; PEP: post-exposure prophylaxis; PHO: Public Health Ontario; PHOL: Public Health Ontario Laboratories; TPH: Toronto Public Health.

All costs are reported in 2015 Canadian dollars and 2015 Euros (CAD 1 = EUR 0.71).

### Effectiveness of outbreak response

This economic evaluation was informed by data derived from the 2015 measles outbreak. The estimated outbreak size with no public health intervention was determined using an existing short-term mathematical model applying the susceptible-infectious-recovered (SIR) framework (transmission model) and using provincial data on the number of susceptible individuals by age group (i.e. population data, vaccination coverage, vaccine effectiveness) (Supplement) [16-18]. Ontario’s two-dose vaccine coverage was estimated to be 89.4% and 94.3% in 7-year-olds and 17-year-olds in 2014-2015, respectively [10]. The high infectivity of measles was taken into account with an R_0_ of 12 to 18. In the transmission model, the population was stratified by age, and each age group had varying inter- and intra-contact rates. Approximate contact rates between age groups were derived from data from a survey conducted in eight high-income European countries [19].

### Disease history

The number of measles cases and contacts in the outbreak were derived from Ontario data provided by the LPHA where the outbreak occurred and have been described previously [9].

Targeted literature searches were conducted to identify the probabilities of short- and long-term measles sequelae. Probabilities for the following health outcomes were included (Table 1): febrile seizure, diarrhoea, otitis media, pneumonia, encephalitis, post-infection encephalomyelitis and subacute sclerosing panencephalitis (SPPE) [20-22]. The probability of long-term sequelae resulting from encephalitis was reported to be 0.250 [23].

Acute measles infection typically requires medical attention including outpatient, inpatient and emergency room visits. The probabilities of healthcare use included 0.775 for outpatient physician visit, 0.250 for emergency room visits and 0.192 for hospitalisation [24].

The case fatality ratio (CFR) of measles varies between locations and epidemiological contexts [25-27]. Canada has not had a case of endemic or imported measles that has resulted in death recently, making it difficult to ascertain a CFR specific to Canada [28-30]. Using CFR data from other high-income countries, the estimated range is from 0.04 to three deaths per 1,000 measles cases [26,31-34]. For this analysis a 0.002 probability of death was assumed.

### Quality of life

Age-specific utility weights for measles infection and measles infection with sequelae (i.e. pneumonia and short- and long-term encephalitis) were derived from Thorrington et al. and Lee et al. [35,36]. Age-specific utilities for the well state were obtained from Mittmann et al. [37]. The utility of being healthy ranged from 0.92 (20 years and older) to 0.94 (0–19 years) [37], and the utility with measles infection ranged from 0.90 (13 years and older) to 0.92 (0–12 years) [35]. The utility for pneumonia infection ranged from 0.82 (adolescent) to 0.91 (adult). The utility of short-term encephalitis is 0.51 and for long-term sequelae of encephalitis is 0.77, based on short- and long-term neurologic complications [36].

### Cost of intervention

#### Local public health agency resource use and cost

Staff salary data for TPH was compiled from data entered into their Incident Management System (IMS) that was initiated specifically for this outbreak. TPH costs also included travel, parking and materials associated with their outbreak response efforts.

Salary data for NRPH involved in the outbreak response was compiled from staff time of nursing staff, programme assistants and health promotion and communication staff, including both regular hours and overtime hours worked.

#### Provincial and federal public health costs

Compensation for unionised PHO staff was based on the estimated number of hours worked on the outbreak and the mid-point of the pay band (which is specified by the union). For non-unionised staff (who do not fall within a specified pay band that would allow estimation of costs), a dollar amount based on time worked on the outbreak was provided by the staff member.

Costs associated with laboratory testing were provided by PHO laboratories and NML, who conducted laboratory tests associated with the measles outbreak. The cost estimates included both staff time and testing costs.

#### Vaccine costs

Prices for vaccines used in publicly funded programmes are not publicly available in Ontario, therefore CAD 20 per dose (EUR 14) of measles-mumps-rubella (MMR) vaccine was assumed. This was based on costs from the United States Centers for Disease Control and Prevention (2015 CAD 25.46/EUR 18) [38] and considered the volume discount that the Ontario MOHLTC may have reasonably achieved.

In this analysis, we included 15,929 doses of MMR vaccine as attributable to the outbreak. The number of doses administered as a result of the outbreak was estimated using vaccine distribution data from the MOHLTC with consideration of the usual distribution during non-outbreak months (which we expect would have been administered routinely) and potential for unused vaccine doses that may have been returned to the ministry.

TPH reported that they administered 13 doses of post-exposure prophylaxis (PEP), and the estimated cost of the anti-measles immunoglobulins (IgG) was CAD 100 or EUR 71 per dose [39].

#### Healthcare costs

The mean cost per hospitalisation (CAD 5,298/EUR 3,761) and emergency department visit (CAD 195/EUR 138) for measles was estimated using health administrative data from the Discharge Abstract Database (DAD) and the National Ambulatory Care Reporting System (NACRS), respectively, for all measles cases in Ontario from 2003 to 2015, excluding non-typical cases (where length of stay was >400 days) [40,41]. The cost of measles treatment in primary care settings was estimated using physician billing codes from the Ontario Health Insurance Plan (OHIP) for all records with a measles diagnosis code from 2001 to 2015 [42], resulting in an average cost of CAD 30 or EUR 21 per physician visit.

### Intervention

The base-case analysis represented the outbreak as observed (intervention) compared with the modified response scenario. In the actual outbreak, there were approximately 18 contacts per case or suspected case, who contributed to the case and contact follow-up activities. The modified response scenario used the following key assumptions: 10% of LPHA costs, 10% of PHO costs, 100% of laboratory costs and no MMR vaccine doses attributable to the outbreak, representing a less intense response from the LPHA (minimal contact follow up). In the hypothetical modified response scenario, we assumed that only high-risk contacts (e.g. immunocompromised persons, susceptible pregnant women, infants younger than 12 months) would be followed up by public health rather than all potential exposed persons in public settings. The modified response also assumed that extra outbreak management measures would not be implemented, including a measles phone hotline and the mailing of notifications to hundreds of potential contacts. The laboratory costs remained unchanged because cases and suspected cases of measles would still be tested. The provincial and local public health costs were reduced to 10% as a conservative estimate because it is likely that some high-risk contact follow-up and case data entry would still take place in a reduced response scenario.

### Sensitivity analysis

One-way sensitivity analyses were conducted on a number of parameters including: vaccine price, number of vaccine doses administered during the outbreak, number of cases prevented, hospitalisation cost per visit, emergency room visit cost per visit, outpatient care cost per visit, utilities, sequelae probabilities and public health response costs (including PHO, LPHA and laboratory costs). Two-way sensitivity analysis was conducted on vaccine price and number of prevented cases.

### Ethics statement

We obtained ethics approval from Public Health Ontario’s research ethics board.

## Results

### Observed and predicted cases

The Ontario measles outbreak comprised 16 cases, 173 suspected cases (later confirmed negative), and 3,369 contacts (Table 2). Based on the short-term model used to predict the final size of the outbreak with no intervention, the number of measles cases ranged from approximately two cases (fewer than the actual outbreak) to 32 cases depending on the number of index cases and their age group (Supplement). For the purpose of this study, the number of cases included to model the modified response scenario was 32 cases, which is the highest number of expected cases (with five index cases aged 5–9 years). Since the cases predicted in the modified response scenario was double the observed cases in the actual outbreak, we also doubled the suspected cases and contacts by age group (Table 2).

**Table 2:** Actual and predicted distribution of cases and contacts by age, measles outbreak Ontario, Canada, 2015

	Actual outbreak^a^			Predicted outbreak, modified response		
	Toronto	Niagara Region	Total	Toronto	Niagara Region	Total
Confirmed cases	10	6	**16**	20	12	**32**
**<5 years**	3	0	**3**	6	0	**6**
**5–19 years**	0	4	**4**	0	8	**8**
**≥20 years**	7	2	**9**	14	4	**18**
Suspected cases	148	25	**173**	296	50	**346**
**<5 years**	54	12	**66**	108	24	**132**
**5–19 years**	38	6	**44**	76	12	**88**
**≥20 years**	56	7	**63**	112	14	**126**
Contacts	1,532	1,837	**3,369**	3,064	3,674	**6,738**
**<5 years**	169	110	**279**	338	220	**558**
**5–19 years**	208	1,617	**1,825**	416	3,234	**3,650**
**≥20 years**	1,155	110	**1,265**	2,310	220	**2,530**

**^a^** Cases, suspected cases and contacts for the 2015 measles outbreak were provided by the affected local public health agencies based on their actual outbreak response.

### Base-case analysis results

Table 3 presents projected health outcomes, costs and incremental cost-effectiveness ratios (ICERs) for the public health response to the 2015 Ontario measles outbreak compared with the modified response scenario. The outbreak response provided a total population health gain of 94,134,922 QALYs (128,803,477 QALYs undiscounted). Few short- or long-term events were estimated to occur: one case of pneumonia, one case of otitis media and one case of diarrhoea occurred, and the model predicted less than one case of encephalitis as a result of measles infection during the outbreak. The total cost of the outbreak response was CAD 1,203,351 (EUR 854,379) during the outbreak year; over a lifetime, there would be an additional cost of CAD 10,141 or EUR 7,200 (CAD 16,274/EUR 11,555 undiscounted) for long-term sequelae (total cost CAD 1,213,491/EUR 861,579; discounted at 1.5%). The majority of the cost (CAD 540,136/EUR 383,497; 45%) was associated with the LPHA response, followed by the cost of distributed MMR vaccine (CAD 318,580/EUR 226,192; 26%). Short-term infection costs (emergency room visits, hospitalisations and physician visits) cost CAD 17,508 (EUR 12,431) during the outbreak year. The public health response to the measles outbreak was expected to have prevented 16 cases of measles, equivalent to approximately 1 discounted QALY (3 undiscounted) gained.

**Table 3:** Base-case results for public health response to the measles outbreak, undiscounted, discounted at 1.5% and discounted at 3%, Ontario, Canada, 2015

	Actual response	Modified response	Difference
Measles cases	16	32	−16
QALYs			
Undiscounted	128,803,476.86	128,803,474.48	2.38
Discounted at 1.5%	94,134,921.78	94,134,920.64	1.14
Discounted at 3%	57,502,634.96	57,502,634.17	0.79
Total cost (CAD; EUR)			
Undiscounted	1,219,625; 865,934	380,972; 270,490	838,653; 595,444
Discounted at 1.5%	1,213,491; 861,579	368,705; 261,781	844,787; 599,799
Discounted at 3%	1,208,502; 858,036	358,727; 254,696	849,776; 603,341
ICER (CAD/QALY; EUR/QALY)			
Undiscounted			352,502; 250,276
Discounted at 1.5%			739,063; 524,735
Discounted at 3%			1,077,334; 764,907

ICER**:** incremental cost-effectiveness ratio; QALY: quality-adjusted life year.

Dollar values rounded to the nearest whole dollar; unrounded values used to calculate ICERs. All costs are reported in 2015 Canadian dollars and 2015 Euros (CAD 1 = EUR 0.71).

The modified response scenario had an expected 32 measles cases, a total cost (costs accrued in the outbreak year and long-term sequelae costs) of CAD 368,705 or EUR 261,781 (CAD 380,645/EUR 270,258 undiscounted) and a total of CAD 94,134,921/EUR 668,357,880 (CAD 128,803,474/EUR 91,450,467 undiscounted) lifetime QALYs. The ICER of the outbreak response was CAD 739,063 or EUR 524,735 per QALY gained (CAD 352,502/EUR 250,276 per QALY gained, undiscounted) compared with the modified response scenario. In order to meet the commonly used cost-effectiveness threshold of CAD 50,000 (EUR 35,500) per QALY gained, the outbreak response would have to prevent 94 cases of outbreak-associated measles.

### Sensitivity analysis

**Figure 2:**
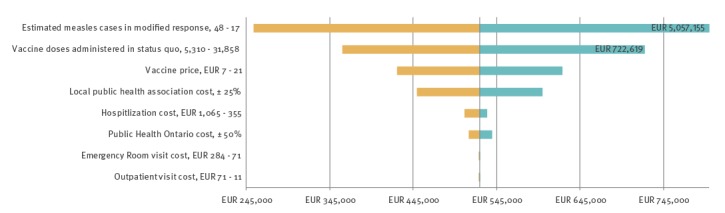
Tornado diagram showing one-way sensitivity analysis for key variables, measles outbreak Ontario, Canada, 2015

ICER: incremental cost-effectiveness ratio; QALY: quality-adjusted life year.

**Figure 3:**
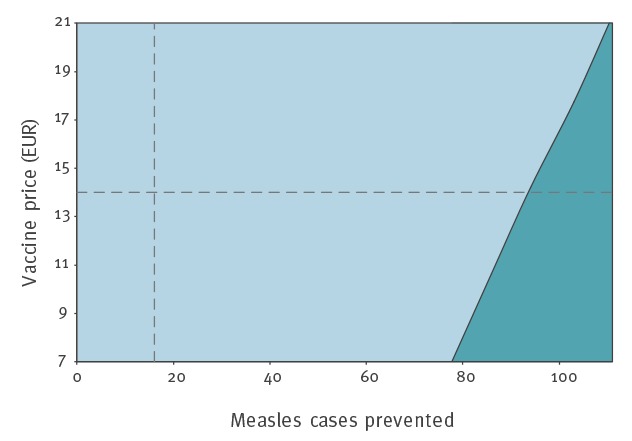
Two-way sensitivity analysis on vaccine price and measles cases prevented for the base-case comparing ‘actual outbreak response’ to a hypothetically reduced ‘modified response’ (discounted at 1.5%), measles outbreak Ontario, Canada, 2015

Results of the one-way sensitivity analysis are shown in Figure 2; variables that were tested but did not result in notable changes in estimated ICER are not reported in the figure. The conclusion was robust to changes in vaccine price, number of doses administered, number of cases prevented and treatment costs (hospitalisation, emergency room visits, and outpatient visits). In a two-way sensitivity analysis on vaccine price and prevented measles cases, the number of prevented cases necessary to reach a CAD 50,000 (EUR 35,500) threshold ranged from 78 (with a CAD 10/EUR 7 vaccine) to 111 (with a CAD 30/EUR 21 vaccine) (Figure 3).

The x-axis represents the range of the ICER when the base-case values are varied. The vertical line represents the base-case ICER (CAD 739,063/EUR 524,735 per QALY gained).

All costs are reported in 2015 Euros (equivalent to 2015 CAD 1.41).

This graph shows the impact on the decision of simultaneous changes in the values of two variables (vaccine price per dose and measles cases prevented by outbreak response) in a region graph. The region graph identifies changes in the preferred strategy using a cost-effectiveness threshold of CAD 50,000 (EUR 35,500) per QALY gained.

Light blue: variable combinations for which ‘modified response’ is the preferred strategy; dark blue: variable combinations for which ‘actual outbreak response’ is the preferred strategy; dotted lines: base-case values.

Overall, from a healthcare payer perspective, Ontario’s public health response during the measles outbreak would not be considered cost-effective under the commonly used cost-effectiveness threshold across reasonable ranges for the variables tested in sensitivity analyses.

## Discussion

Our findings indicate that the public health response to the measles outbreak in 2015 had an ICER of CAD 739,063 (EUR 524,735) per QALY gained compared with the modified response, and would have had to prevent 94 cases of measles in order to reach a CAD 50,000 (EUR 35,500) cost-effectiveness threshold. Since the number of cases, probability of sequelae and healthcare costs were low, changes in healthcare costs over a reasonable range did not impact the number of cases to prevent in order to reach the cost-effectiveness threshold. Given the high measles vaccine coverage in Ontario [10], it is unlikely that importation of measles and subsequent transmission would cause an outbreak large enough for the outbreak response to prevent enough cases to be considered cost-effective. Trends over the last 10 years in Ontario support this, with the largest number of measles cases being 58 cases in 2008 and 22 cases in 2014 [2]. A measles outbreak of a much larger magnitude than is typically observed would need to occur before the outbreak response would be cost-effective. However, the generalisability of these results is low in settings in which measles has not been eliminated, such as some countries in Europe, where recent measles outbreaks have been larger and many key parameters used in the model may differ (e.g. vaccine coverage).

Some scenarios exist in which the outbreak response may be found to be more favourable. Firstly, an outbreak response that focused on under-immunised communities may provide more benefit than a broad outbreak response for the entire population. In addition, given that 26% of costs of the outbreak response were associated with increases in MMR vaccine distribution, it is possible that a lower vaccine price may enable a more favourable implementation of the public health response.

The results of the current analysis should not be directly applied to jurisdictions that have not yet achieved measles elimination status (which includes many countries in the European Union who have been experiencing large numbers of measles cases [43]). Given the global context of measles elimination, and the fact that all six regions of the World Health Organization (WHO) have a measles elimination goal [44], this work demonstrates that the measles outbreak response is unlikely to be good value for money in the increasing number of jurisdictions which have succeeded in eliminating measles, including those in the WHO European Region. Further, given that the economic model relied heavily on real-world data from Ontario, the deterministic sensitivity analysis results are likely to be the most useful results to apply to other jurisdictions in order to identify which parameters impact the economic model most (rather than probabilistic sensitivity analysis which would not be as transferable). In this case, the most influential parameter was the number of measles cases that occurred in the hypothetically reduced modified response scenario, which was more cost-effective with increasing case numbers. This suggests that in European jurisdictions experiencing high numbers of measles cases, results of a similar cost-effectiveness analysis could be quite different from the results presented for the Ontario context.

Our study had several limitations. The economic evaluation of public health interventions in the context of disease elimination and eradication poses particular challenges because of the global nature of elimination efforts and need for enhanced disease control activities. Our economic evaluation did not consider the potential future benefits of global measles eradication, which would result in reduced measles control efforts and eliminate the need for vaccination in the future. Indeed, there is no agreed methodology for making an economic case for sustaining a level of public health response at the same level as that needed to achieve measles elimination during the subsequent post-verification period. That post-verification period, during which importations continue to be a risk while measles circulates in other countries, is potentially prolonged with no global eradication goals. Given the low number of measles cases expected in an elimination setting, and a declining number as more regions around the world achieve measles elimination, it is unlikely that public health interventions would be considered cost-effective at currently accepted thresholds, underscoring the importance of setting a goal for global measles eradication in the near future.

This evaluation did not capture the benefit from potential local increases in MMR vaccine coverage that may have resulted from publicity about the outbreak. Given the increase in vaccine doses distributed to the affected regions during the outbreak by the MOHLTC, it is possible that Ontario will experience higher vaccine coverage that may prevent measles transmission in the future. The magnitude of these benefits would need to be considerable to shift the conclusions of our model. Assumptions were made about some variables that were difficult to obtain. Vaccine prices are not publicly available in Ontario, so we made assumptions about the bulk purchasing discount that may have been achieved. While we had data from the MOHLTC regarding the number of MMR vaccine doses distributed to TPH and NRPH, we were unable to determine exactly how many doses were administered as a direct outcome of the outbreak, how many were given as part of routinely scheduled immunisation and how many were stored and used in future months outside of the outbreak. This evaluation did not include adverse events following immunisation (AEFI) in the decision-analysis model because of the low probability of serious AEFIs occurring. Since this analysis considered approximately 16,000 MMR doses, the very small probability of a serious event occurring was unlikely to change the conclusions of the model, i.e. the current containment strategy would provide even less value than a modified (reduced) response.

The mathematical model used to predict outbreak outcomes with no response (transmission model) had some limitations. Thanks to the high vaccine coverage in Ontario, the size of the susceptible population is small, leading to uncertainty in the estimation of predicted cases because of stochasticity. However, the transmission model was based on a scenario where no intervention took place, whereas our analysis included some response that probably would have prevented some cases. Alternative structures were considered for the transmission model (e.g. network structure and household-structured models) but these model structures are complex and, for the purposes of this economic evaluation, were not necessary. Rather, to account for uncertainty in the transmission model we conducted sensitivity analyses around the anticipated number of measles cases given no outbreak response in the decision-analysis model. In addition, the heterogeneity of measles coverage across Ontario could result in larger than predicted measles outbreaks if the index case occurred in a region with lower vaccine coverage than used in this transmission model. For example, low vaccine coverage in a community in Oxford County, Ontario resulted in a mumps outbreak of 324 cases in 2008, significantly larger than usually seen in Ontario (with a range from 10 to 107 cases per year between 2005 and 2016) [45,46]. According to the Centers for Disease Control and Prevention, these pockets pose a threat in the United States as well [47]. Despite uncertainty in the transmission model, it was consistent with past numbers of measles cases in Ontario, which tend to be small, and offered a conservative estimate for use in the decision-analysis model.

The strengths of this study include that the decision-analysis model was based on data from an actual measles outbreak, parameters were tested in sensitivity analyses and input data was carefully selected to be representative of the Ontario population. The transmission model used to predict outbreak outcomes with no response was robust and included Ontario-specific data on vaccine coverage and population distribution.

To the best of our knowledge, no prior economic evaluation of a measles outbreak response has been conducted in Canada. This is an important context to evaluate given the elimination status of measles in our jurisdiction. In the Netherlands, there has been a study on the economic costs of a measles outbreak in 2013 and 2014, however, this study did not include a cost-effectiveness analysis [48]. The outbreak in the Netherlands with 2,700 reported measles cases was much larger than the one in Ontario, making comparison between the two challenging [9,48]. The Dutch study reported that the highest proportion of costs were associated with the municipal public health services (responsible for registering cases and providing advice), followed by hospitalisation costs [48]. The highest costs in Ontario were also associated with case and contact management at the LPHA level.

This economic evaluation may be helpful to other jurisdictions with similar healthcare systems, elimination status and high vaccine coverage. Measles has been eliminated in the Americas [7], which could mean many other jurisdictions have similar MMR vaccine coverage and population at risk of measles transmission. The results of this economic evaluation should be used in conjunction with other relevant evidence in order to evaluate Ontario’s current practices during measles outbreaks.

## Conclusions

Ontario’s measles outbreak response exceeds the generally accepted cost-effectiveness thresholds; to be considered cost-effective, it would require more measles cases to be prevented than are typically experienced during Ontario outbreaks. Given Ontario’s high vaccine coverage and Canada’s measles elimination status, the current outbreak response protocols in Ontario may not be the most efficient use of public health resources from a healthcare payer’s perspective. These findings should be balanced against benefits of the measles outbreak response that we could not include in the model, notably increased vaccine coverage and the contribution towards maintaining elimination status.

## Supplementary Data

176000 Supplement
