# Supplementary material for: Cost-effectiveness of measles control during elimination in Ontario, Canada, 2015
Source: Euro Surveill. 2019 Mar 14;24(11):1800370. doi: 10.2807/1560-7917.ES.2019.24.11.1800370 (PMC6425553; doi:10.2807/1560-7917.ES.2019.24.11.1800370)
Supplement: Supplement [file 18-00370_RAMSAY_Supplement.pdf]

Supplement 1 –
Measles in Ontario: Short Term Model

Elena Aruffo, Alexandra Teslya, Jane Heffernan

This supplementary material is hosted by *Eurosurveillance* as supporting information alongside the article ``Cost-effectiveness of measles control during elimination in Ontario, Canada, 2015`` on behalf of the authors who remain responsible for the accuracy and appropriateness of the content. The same standards for ethics, copyright, attributions and permissions as for the article apply. *Eurosurveillance* is not responsible for the maintenance of any links or email addresses provided therein.

Abstract

We report the final size of a measles epidemic initiated in different age groups of a population. The population size is determined by the number of susceptible individuals in each age class reported, as per Public Health Ontario data. We built a short-term model using a Susceptible- Infected-Recovered (SIR) framework to model measles spread in the population. The epidemic final size varies depending on the number of initial cases introduced into the population, and the age group at which these individuals belong to. By seeding the epidemic with individuals aged 5-9 we find that the final size of the epidemic ranges from 5 cases (when one infected individual is introduced into the population) to 24 cases (when 5 individuals are introduced into the population). A final size of 13-14 cases requires the introduction of 2 individuals aged 5-9, that are independent of each other.

1 Initial data and the model

PHO provided data, giving the number of susceptible individuals by age group. The data considered the uptake of vaccine (0,1, 2 shots) and overall vaccine efficacy. The data divided the susceptible population into 46 compartments: 0 − 6 months, 1 year, 2 years, . . . , 45 years. The objective of this modelling study was to examine the final size of the epidemic by infectious case introduction (age and number of individuals).

The following are the modelling assumptions:

1. The basic reproductive number R_0 for measles is 18.
2. The dynamics of measles are modelled using Susceptible-Infectious-Recovered framework.
3. The population dynamics are spatially homogeneous;
4. Since the model is short term (duration of an epidemic) no demographic processes (birth, death etc.) were incorporated;
5. The population is delineated by age. Each group has varying inter and intra contact rates,

as well as the initial number of susceptible people.

6. The infection transmission rate is a product of contact rate and infectivity. Infectivity is taken to be the same over all infectious groups;

7. To approximate contact rates between different age groups we use a survey given in [1]. Data relevant to the Netherlands (Table S1) was chosen. Note that the grouping in this matrix is coarser than the susceptibility data provided by PHO. We will deal with this in two ways. We
will develop a model where the size of contact matrix will fit the length of the susceptibility vector, by making subgroups in each age group of the contact matrix have the same contact rates (for example, we will break group 0-4 years into 6 groups with similar contacts. The second approach involves grouping age groups in the PHO provided data into groups of people that correspond to the matrix in [1].

8. There are no disease induced deaths.

9. The length of infectious period is 7 days.

The following model is designed for a population divided into n groups. This division is with respect to age. The dynamics of the system can be described by the following set of equations:

$$\dot{S}_i(t) = -S_i \sum_{j=1}^{n} \frac{\beta_{ij}}{N} I_j \ , \tag{1a}$$

$$\dot{I}_i(t) = S_i \sum_{j=1}^{n} \frac{\beta_{ij}}{N} I_j - \gamma I_i \ , \tag{1b}$$

$$\dot{R}_i(t) = \gamma I_i \ , \tag{1c}$$

with $i = 1 \ldots n$. X_i denotes a person in age group i. S denotes susceptible, I infectious, R recovered and N is the total population. Observe that if we "stretch" the contact matrix in Table S1 to match susceptibility data, then $n = 46$ and the correspondence between indexing of groups and the actual age is $i = 1 \rightarrow 6 - 12$ month, $i = 2 \rightarrow 1$ year, $i = 3 \rightarrow 2$ years,. . . $i = 46 \rightarrow 45$ years. Finally, note that, we divide each entry of Table S1 by 5 to obtain the contact rates between the individuals in 46 groups. If, on the other hand, the susceptibility data is "compressed", then $n = 10$, where $i = 1 \rightarrow 0 - 4$ years, $\ldots i = 9 \rightarrow 40 - 44$ years, $i = 10 \rightarrow 45$ years.

The initial contact rate matrix for Netherlands is given by Table S1. We "stretch" Table S1 to obtain Table S2. (Note that this is a very rough approximation of the contact matrix over five age groups in each category of Table S1. The meaning and value of the rest of parameters is as follows:

- γ is the rate of leaving infectious compartment, where $\gamma = 1/7$ days.
- $\beta_i j = p \times c_{ij}$, where p the infectivity and $c = [c_{ij}]$ is the contact rate between people in group i with people in group j. We use Netherlands data for contacts. We calculate p as follows. If we assume that the contact number between each group is the same average number c and therefore $\beta = c \times p$ and $R_0 = \frac{\beta}{\gamma}$. Therefore, $p = \frac{18\gamma}{c}$. We find the average amount of contacts a person in each age group makes per day. Then, the average contact rate is a weighted average

(by the age group size). To calculate an average number of contacts we only consider these groups that according to PHO data have susceptible people in them, i.e. people younger than age of 46 years and not including babies aged 0-6 month. The contact data for Netherlands is given by Table S1. If we stretch contact matrix to fit susceptibility data we obtain c = 1.4789 and p = 1.7387 and, while in the model where susceptible data is compressed to match the contact matrix c= 1.8670 and p = 1.3773.

	Age group of participant (years)									
Age of contact (years)	0-4	5-9	10-14	15-19	20-24	25-29	30-34	35-39	40-44	45-49
0-4	3.50	1.71	0.25	0.19	0.33	0.57	1.00	0.92	3.00	0.00
5-9	2.47	11.88	1.56	0.19	0.00	0.50	1.25	1.62	1.14	0.71
10-14	0.32	1.06	10.97	2.50	0.08	0.00	1.50	1.00	0.71	0.93
15-19	0.15	0.24	1.44	9.19	0.83	0.29	1.25	0.08	0.57	0.79
20-24	0.24	0.18	0.16	1.25	2.42	0.57	0.75	0.85	2.29	0.71
25-29	0.21	0.88	0.13	0.44	1.58	1.86	2.25	0.62	0.86	1.21
30-34	1.26	0.71	0.25	0.44	2.67	1.00	3.75	1.00	1.71	0.86
35-39	1.15	1.53	0.78	0.75	1.58	0.86	3.00	3.92	1.29	1.79
40-44	0.53	1.76	1.56	0.69	0.67	1.07	2.13	3.08	1.86	2.50
45-49	0.24	0.47	0.94	1.38	0.92	0.50	1.63	1.69	1.29	2.21

Table S1: Contact rates between age groups as surveyed by [1] in Netherlands

	Age group of participant								
Age of contact	6-12 months	1 year	...	4 years	5 years	...	9 years	...	45 years
6-12 months	0.7	0.7	...	0.7	0.342	...	0.342	⋮	0.00
1 year	0.7	0.7	...	0.7	0.342	...	0.342	⋮	0.00
⋮	⋮	⋮	⋮	⋮	⋮	⋮	⋮	⋮	⋮
4 years	0.7	0.7	...	0.7	0.342	...	0.342	⋮	0.00
5 years	0.494	0.494	...	0.494	2.376	...	2.376	...	0.142
⋮	⋮	⋮	⋮	⋮	⋮	⋮	⋮	⋮	⋮
9 years	0.494	0.494	...	0.494	2.376	...	2.376	...	0.142
⋮	⋮	⋮	⋮	⋮	⋮	⋮	⋮	⋮	⋮
45 years	0.048	0.048	...	0.048	0.094	...	0.094	...	0.442

Table S2: Contact rates between age groups as surveyed in Netherlands and extended to accommodate the granularity of susceptibility data

2 Results

2.1 "Stretched Contact Matrix Approach"

We have seeded the infection one compartment at a time, using an increasing number of index cases. Table S3 summarizes our results for the model.

2.2 "Clustered Contact Matrix Approach"

As already mentioned in the model description, we have grouped the age classes into 10 different groups.

As before, we have seeded the infection one compartment at a time, with a different number of infected individuals. Table S4 lists the final size of the epidemic as the number of initial infecteds, and the age of the initial infecteds varies.

Age	Δ Susceptible	Age	Δ Susceptible	Age	Δ Susceptible	Age	Δ Susceptible	Age	Δ Susceptible
6-12 m	3.11	10 y	2.48	20 y	1.15	30 y	2.18	40 y	2.13
1 y	3.11	11 y	2.48	21 y	1.15	31 y	2.18	41 y	2.13
2 y	3.11	12 y	2.48	22 y	1.15	32 y	2.18	42 y	2.13
3 y	3.11	13 y	2.48	23 y	1.15	33 y	2.18	43 y	2.13
4 y	3.11	14 y	2.48	24 y	1.15	34 y	2.18	44 y	2.13
5 y	4.7	15 y	1.62	25 y	1.22	35 y	2.56	45 y	1.31
6 y	4.7	16 y	1.62	26 y	1.22	36 y	2.56		
7 y	4.7	17 y	1.62	27 y	1.22	37 y	2.56		
8 y	4.7	18 y	1.62	28 y	1.22	38 y	2.56		
9 y	4.7	19 y	1.62	29 y	1.22	39 y	2.56		

Table S3: Final size of the epidemic given a single infected in each age group. The final size ranges from 1 to 5 cases. If more infecteds are introduced into the population, the final size will grow.

1 infected individual		5 infected individuals	
Age group	Δ Susceptible	Age group	Δ Susceptible
0-4 y	4.22	0-4 y	21.11
5-9 y	6.48	5-9 y	32.39
10-14 y	3.29	10-14 y	16.45
15-19 y	2.09	15-19 y	10.45
20-24 y	1.49	20-24 y	7.49
25-29 y	1.61	25-29 y	8.07
30-34 y	2.89	30-34 y	14.45
35-39 y	3.41	35-39 y	17.06
40-44 y	2.84	40-44 y	14.21

Table S4: Variations in susceptible population as the placement of 1.5 index cases changes. The final size ranges from 1 to 5 cases of measles. The final size ranges from 1.72 cases to almost 7 cases when only 1 initial infected is introduced into the population. It ranges from 7 to 33 cases if 5 infected individuals are introduced into the population.

3 Conclusions

The age group location of the initial case, and the number of initial cases affect the final size (as expected). In the models that we considered, initial infecteds aged 5-9 resulted in the largest epidemic final size. If one initial infected aged 5-9 years old, the expected final size of the epidemic is almost 7 cases. An expected final size of 13-14 cases requires two initial infected individuals aged 5-9, that are independent of each other. A stochastic model, if developed, would provide the variability around these expected values.

A more refined contact matrix, and one that is applicable to the Canadian population is needed to provide better estimates.

References

[1] Mossong J, Hens N, Jit M, et al. Social contacts and mixing patterns relevant to the spread of infectious diseases. PLoS Med. 2008
